# Infection with SARS-CoV-2 causes abnormal laboratory results of multiple organs in patients

**DOI:** 10.18632/aging.103255

**Published:** 2020-06-01

**Authors:** Ai-Ping Yang, Hui-Ming Li, Wen-Qiang Tao, Xue-Jing Yang, Min Wang, Wen-Juan Yang, Jian-Ping Liu

**Affiliations:** 1 Department of Clinical Laboratory, Zhejiang Xiaoshan Hospital, Hangzhou, Zhejiang Province, China; 2 Department of Clinical Laboratory, The First Affiliated Hospital of Nanchang University, Nanchang, Jiangxi, China; 3 Department of Critical Care Medicine, The First Affiliated Hospital of Nanchang University, Nanchang, Jiangxi, China; 4 Department of Clinical Laboratory, The First Affiliated Hospital of Zhejiang Chinese Medical University, Hangzhou, Zhejiang Province, China

**Keywords:** coronavirus, COVID-19, lymphopenia, inflammatory cytokine, D-dimer

## Abstract

Aim: To evaluate the clinical value of abnormal laboratory results of multiple organs in patients with coronavirus disease 2019 (COVID-2019) and to help clinicians perform correct treatment.

Results: Elevated neutrophil-to-LYM ratio (NLR), D-dimer(D-D), interleukin (IL)-6, IL-10, IL-2, interferon-Y, and age were significantly associated with the severity of illness. However, significant and sustained decreases were observed in the LYM subset (p<0.05). D-D, T cell counts, and cytokine levels in severe COVID-19 patients who survived the disease gradually recovered at later time points to levels that were comparable to those of mild cases. Second, D-D increased from 0.5 to 8, and the risk ratio increased from 2.75 to 55, eventually leading to disseminated intravascular coagulation. Moreover, the acute renal function damage occurred earlier than abnormal heart and liver functions (p<0.05).

Conclusions: The degrees of lymphopenia and proinflammatory cytokine storm were higher in severe COVID-19 patients than in mild cases. The degree was associated with the disease severity. Advanced age, NLR, D-D, and cytokine levels may serve as useful prognostic factors for the early identification of severe COVID-19 cases.

Methods: Peripheral blood samples were collected from 93 confirmed COVID-19 patients. The samples were examined for lymphocyte (LYM) subsets by flow cytometry and cytokine profiles by specific immunoassays. The receiver operating characteristic curve was applied to determine the best diagnostic thresholds for laboratory results, and principal component analysis was used to screen the major risk factors. The prognostic values were assessed using the Kaplan–Meier curve and univariate and multivariate COX regression models.

## INTRODUCTION

Coronavirus is a large virus family known to cause multiple system infections in various animals and mainly respiratory tract infections, such as severe acute respiratory syndrome (SARS) [1–3] and the Middle East respiratory syndrome (MERS) [4], in humans. Although the clinical characteristics of coronavirus disease 2019 (COVID-19) have been broadly defined [5], an outline of the most representative laboratory abnormalities observed in patients with COVID-2019 is still incomplete [6–7]. Laboratory medicine plays an essential role in the early detection, diagnosis, and management of numerous diseases [8]. COVID-2019 is no exception to this rule. Nevertheless, the role of laboratory diagnostics extends beyond etiological diagnosis and epidemiologic surveillance, whereby in vitro diagnostic tests are commonly used for assessing disease severity, defining the prognosis, patient follow-ups, treatment guide, and therapeutic monitoring [9]. Diagnostics identify the defining laboratory results and clinical characteristics with high precision and unravel the risk factors associated with mortality.

Lymphopenia and inflammatory cytokine storm are typical laboratory abnormalities observed during highly pathogenic coronavirus infections, such as SARS coronavirus (SARS-CoV) and MERS coronavirus (MERS-CoV) infections; these abnormalities are believed to be associated with disease severities [10]. Severe inflammatory responses contribute to the weakening of the adaptive immune response, which results in an imbalanced immune response and COVID-19. Therefore, circulating biomarkers that can represent the status of inflammation and immunity are recognized as potential predictors for the prognosis of COVID-19 patients [11]. Recent studies have also reported decreases in the lymphocyte (LYM) counts in the peripheral blood and increases in serum inflammatory cytokine levels in COVID-19 patients [12]. However, how different LYM subsets and the kinetics of inflammatory cytokines change in the peripheral blood in COVID-19 remain unclear. In this study, the changes in LYM subsets and cytokines profiles in the peripheral blood of COVID-19 patients with distinct disease severities were longitudinally characterized.

## RESULTS

### Results of white blood cell (WBC) count, LYM subset, and demographics of the study subjects

Table 1 showed the demographics and clinical characteristics of the study subjects. The proportion of randomly selected severe cases, including critical illness, was 25.8%. The average age of those was 58 years old, as well as 42 years old of non-severe patients. The age and WBC count, NLR, LYM–monocyte (MON) ratio, platelet-to-LYM ratio, CRP, d-NLR, and D-dimer (D-D) of severe ill patients were significantly higher than those of non-severe patients (p<0.01). By contrast, the results of CD3+, CD3+CD4+, CD3+CD8+, CD56+CD16+, and CD3-CD19+ were notably low (p<0.01). However, no significant difference was observed in terms of gender, Fib, albumin-to-fibrin ratio, and CD4+/CD8+ (p>0.05).

**Table 1:** Results of WBC count, lymphocyte subset and demographic in the study subjects.

**Laboratory results**	**Total**	**non-sever (n=69)**	**Severe (including critical illness) (n=24)**	**P-value**
age(M±SD)	46.4±17.6	42.1±18.6	57.9±11.8	<0.05
sex±M/F±	56/37	38/31	18/6	0.135
WBC(M±SD)	6.9±3.9	6.4±2.4	9.1±5.6	<0.01
LYM	1.04±0.64	1.17±0.63	0.65±0.54	<0.01
NEU	5.38±3.6	4.55±0.21	7.73±5.4	<0.01
MON	0.43±0.46	0.41±0.2	0.5±0.84	<0.05
NLR(M±SD)	10.8±15.6	4.8±3.5	20.7±24.1	<0.01
d-NLR(M±SD)	5.07±5.5	3.3±1.9	9.8±7.8	<0.01
LMR(M±SD)	3.42±4.6	4.1±6.0	2.1±1.6	<0.01
PLR(M±SD)	255.8±226.1	176.7±84.2	436.5±329.2	<0.01
CRP(M±SD)	33.8±48.4	20.1±24.5	53.9±60.1	<0.01
CD3+	629.4±489.4	763.8±483.3	222.2±195.2	<0.01
CD3+CD4+	370.6±264.3	448.7±254.9	132.6±98.5	<0.01
CD3+CD8+	219.8±209.3	264.6±217.4	83.9±97.2	<0.01
CD4+/CD8+	2.06±0.97	2.01±0.98	2.0±0.97	0.754
CD56+CD16+	148.7±132.3	169.3±141.3	85.9±76.7	<0.01
CD3-CD19+	124.8±103.9	141.3±111.2	75.2±53.7	<0.01
D-dimer	3.2±8.1	0.54±0.42	16.6±23.1	<0.01
Alb	38.6±6.9	41.4±5.8	31.9±4.4	<0.05
Fib	3.6±1.3	3.8±1.2	3.2±1.4	0.179
AFR	12.2±5.7	12.2±5.1	12.5±7.4	0.585

Albumin(Alb), Fibrin(Fib), Albumin-to-Fibrin (AFR), white blood count cell(WBC), neutrophil-to-lymphocyte ratio (NLR), derived neutrophil-to-lymphocyte ratio (d-NLR) (neutrophil count divided by the result of white cell count minus neutrophil count), platelet-to-lymphocyte ratio (PLR) and lymphocyte-to-monocyte ratio (LMR), C-reactive protein(CRP), lymphocyte (LYM), Neutrophils (NEU), Monocyte (MON).

### Results of clinical characteristics of the study subjects

All patients had no contact with wild animals. However, 27.8% (26/93) of the patients recently traveled to Wuhan, and 73.1% (68/93) of those had contact with people from Wuhan. Fever and cough were the first and most common symptoms before admission. A total of 50 (53.7%) patients in both groups had co-morbidities, including diabetes (22.5%; 21/93), hypertension (24.7%; 23/93), hepatitis B (11.8%; 11/93), abnormal liver function (13.9%; 13/93), heart disease (13.9%; 13/93), and renal dysfunction (10.7%; 10/93) (Table 2). A total of 70.8% of severe case patients and 79.7% of mild case patients had fever. Meanwhile, no significant difference was observed in the degrees of temperature (p=0.37), fatigue (p=0.213), cough (p=0.496), pharyngalgia (p=0.748), dizziness (p=0.109), headache (p=0.831), chest pain (p=0.456), vomiting (p=0.762), diarrhea (p=0.999), heart disease (p=0.663), and abnormal liver function (p=0.659) between the two groups (Table 2). The severe case patients showed significantly high frequencies in the occurrence of diabetes (p<0.01), hypertension (p<0.01), renal dysfunction (p<0.05), chill (p<0.05), shivering (p<0.05), sputum production (p<0.01), and nausea (p<0.01) (Table 2).

**Table 2:** Baseline characteristics of patients infected with COVID-2019.

**Baseline variables**	**Total(n=93)**	**non-sever (n=69)**	**Severe (including critical illness)(n=24)**	**P-Value**
Wuhan exposure(%)	29 (31.1)	21 (30.4)	9 (37.5)	0.524
co morbidities (%)	50 (53.7)	29 (42.1)	21 (87.5)	<0.01
Diabetes	21 (22.5)	8 (11.6)	13 (54.2)	<0.01
Hypertension	23 (24.7)	7 (10.1)	16 (66.8)	<0.01
hepatitis B	11 (11.8)	7 (10.1)	4 (16.7)	0.409
Heart disease	13 (13.9)	4 (5.8)	9 (37.5)	<0.01
Renal dysfunction	10(10.7)	2 (2.9)	8 (33.3)	<0.05
Abnormal liver function	13 (13.9)	9 (13.0)	4 (16.7)	0.659
others	5 5.4)	3 (4.3)	2 (8.3)	0.456
Signs and symptoms				
Fever	72 (77.4)	55 (79.7)	17 (70.8)	0.37
Chill	22 (23.6)	12 (17.4)	10 (41.6)	<0.05
Shivering	11 (11.8)	5 (7.2)	6 (25)	<0.05
Fatigue	60 (64.5)	42 (60.8)	18 (75)	0.213
Cough	67 (72.1)	51 (73.9)	16 (66.7)	0.496
Sputum production	44 (47.3)	25 (36.2)	19 (79.1)	<0.01
Pharyngalgia	10 (10.7)	7 (10.1)	3 (12.5)	0.748
Dizziness	17 (18.3)	10 (14.5)	7 (29.1)	0.109
Headache	18 (19.3)	13 (18.8)	5 (20.8)	0.831
Chest tightness	28 (30.1)	17 (24.6)	11 (45.8)	0.051
Chest pain	6 (6.5)	3 (4.4)	2 (8.3)	0.456
Shortness of breath	5 (5.4)	2 (2.9)	3 (12.5)	0.072
Nausea	10 (10.7)	4 (5.8)	6 (25)	<0.01
Diarrhoea	1 (1.1)	1 (1.5)	0	0.999
Vomiting	3 (4.3)	2 (2.9)	1 (4.2)	0.762

### Analysis of inflammatory cytokine levels in the serum of COVID-19 patients

A previous study demonstrated the changes in the levels of inflammatory cytokines, such as IL-2, IL-7, IL-10, and tumor necrosis factor (TNF)-α, in the serum of COVID-19 patients [4]. Therefore, the changes in inflammatory cytokine levels, including IL-2 and IL-12P70, were further characterized in the serum of our patient cohort. The severe case patients showed significantly high levels of IL-2 (p<0.05), IL-6 (p<0.01), IL-8 (p<0.05), and IL-10 (p<0.01) (Table 3). No significant difference was observed in the degrees of IL-5, IFN-α, IL-1β, IFN-γ, IL-17, IL-4, and IL-12P70 between the two study groups (Table 3). The laboratory reference values for each cytokine are as follows: IL5 ≤ 3.1 pg/mL, IFN-α≤ 8.5 pg/mL, IL-2 ≤ 7.5 pg/mL, IL-6 ≤ 5.4 pg/mL, IL-1β≤ 12.4 pg/mL, IL-10 ≤ 12.9 pg/mL, IFN-γ≤ 23.1 pg/mL, IL-8 ≤ 5.6 pg/mL, IL-17 ≤ 21.4 pg/mL, IL-4 ≤ 8.56 pg/mL, and IL-12P70 ≤ 3.4 pg/mL.

**Table 3:** Results of inflammatory cytokine levels in the serum of COVID-19 patients.

**Baseline Variables**	**Total (n=93) Mean (Min-Max)**	**non-sever (n=69)**	**Severe (including Critical illness) (n=24)**	**P-Value**
IL-5	2.39(0.2-40.3)	1.99(0.2-10.4)	2.95(0.24-40.3)	0.438
IFN-a	2.31(0.84-22.5)	2.03(0.96-4.83)	2.69(0.84-22.5)	0.617
IL-2	2.21(0.74-25.4)	1.74(0.74-4.76)	2.88(1.23-25.36)	<0.05
IL-6	26.5(0-1197.7)	6.91(0-109.5)	54.1(0-1197.7)	<0.01
IL-1B	14.6(0-121.5)	14.1(0-49.7)	15.5(0-121.5)	0.398
IL-10	3.27(0.96-39.5)	2.81(0.08-10.2)	4.81(1.15-39.5)	<0.01
INF-Y	6.32(0-150.9)	4.46(0.08-67.1)	8.96(0-150.9)	0.218
IL-8	108.3(0-3979.2)	36.1(0-454.3)	210.4(0.57-3979.2)	<0.05
IL-17	1.67(0.17-10.6)	1.56(0.17-10.3)	1.84(0.79-10.6)	0.399
IL-4	1.47(0.53-7.14)	1.51(0.53-7.14)	1.42(0.61-3.56)	0.804
IL-12P70	0.93(0-4.87)	0.85(0-4.5)	1.04(0-4.87)	0.44
TNF-a	19.8(0-1065)	8.29(0-54.4)	35.9(0-1065)	0.577

### Acute heart, liver, and kidney function damage in severe COVID-19 patients

**Figure 1:**
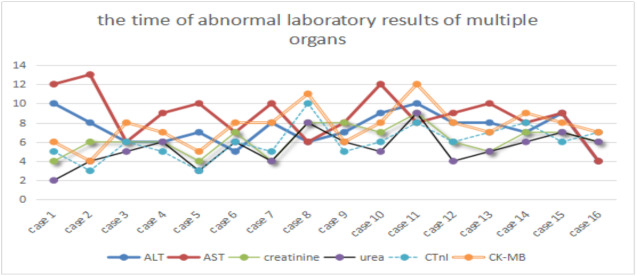
**Time of abnormal laboratory results of multiple organs.** ALT and AST, urea and creatinine, CTnI and CK-MB represent acute liver, renal and heart dysfunctions, respectively. X-axis represents the case number, whereas Y-axis denotes the time of abnormal laboratory results.

Among the 24 severe COVID-19 patients, only 16 cases were selected because of their complete electronic medical records, subsequently, the abnormal laboratory results of multiple organs, such as kidney (creatinine and urea), liver (alanine aminotransferase and aspartate aminotransferase), and heart (High sensitivity troponin T and creatine kinase-MB), were further analyzed. The laboratory results of acute renal function injury (4.94±1.69) occurred earlier than those of liver (7.81±1.86) and heart functions (6.19±1.83). One-way analysis of variance showed a statistically significant difference in laboratory results (p<0.01) between kidney and heart (p=0.056), kidney and liver (P<0.01), and heart and liver functions (P=0.014). However, several patients presented opposite trends. Further checking of the electronic medical record revealed that these patients were infected with hepatitis B and other common diseases, such as liver fibrosis, cyst, fatty liver, and cirrhosis (Figure 1).

### Prognostic factors for the identification of severe COVID-19 cases

**Figure 2:**
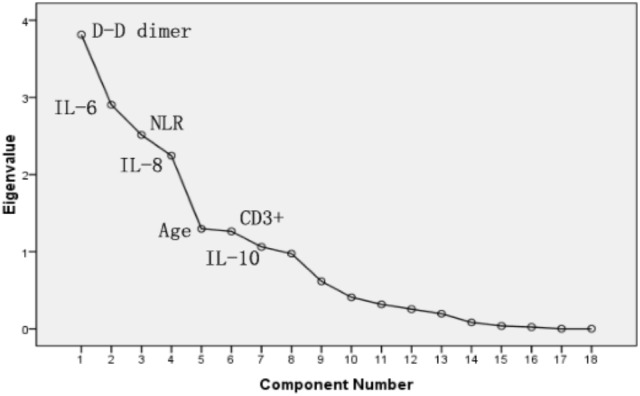
**PCA Sreen point.**

**Figure 3:**
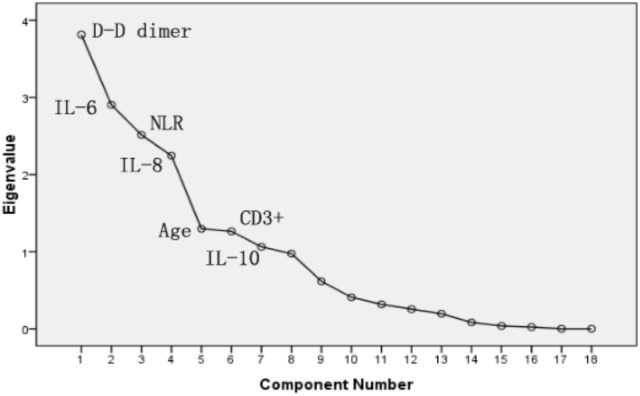
**Receiver operating curve analysis used to identify patients with severe or non-severe cases of COVID-2019.**

PCA was performed by SPSS package “factor analysis” to identify correlated variables for distinguishing severe patients from mild case patients (Figure 2). The seven most contributing variables, namely, D-D, IL-6, IL-8, and NLR with a score of more than 2. CD3+, IL-10 and age with a score of more than 1, which were selected as potential prognostic factors for further detailed statistical analysis. To assess the diagnostic value of the seven selected parameters, we calculated the ROC curve and area under the ROC curve (AUC) by SPSS package (Figure 3). The results of this analysis identified D-D with a higher AUC (0.958) than IL-6 (0.795), NLR (0.789), IL-8 (0.774), age (0.728), and IL-10 (0.717). With values of 49.5 for age, 3.3 for NLR, and 2.1 for D-D dimer, CD3+could not be used as a potential diagnostic biomarker for subsequent analysis with its AUC < 0.50. Meanwhile, the results for IL-10 in severe case patients were statistically higher than those in non-severe patients. However, the average results of both groups were within the reference range (IL-10≤12.9 pg/mL). The further univariate analysis including five factors, such as IL-6, IL-8, D-D, age, and NLR, was used to calculate the odds ratios (ORs) between the severe and non-severe case groups. The results were obtained for NLR (OR: 4.6, 95% Cl: 1.242–17.80), IL-6 (OR: 6.625, 95% Cl: 2.398–18.304), IL-8 (OR: 6.881, 95% Cl: 2.453–19.298), and age (OR: 4, 95% Cl: 1.493–10.714) with our patient cohort as predictive factors for severe COVID-19 (Table 4). D-D increased from 0.5 to 8. The risk ratio between severe and non-severe group increased from 2.75 to 55 and eventually leading to disseminated intravascular coagulation (DIC) (y = 7.0651x - 0.4251, R^2^ = 0.9893).

**Table 4:** The OR in each of the NLR, IL-6, IL-8, age, D-D dimer and double D-D dimer.

	**OR**	**95%CI**		**OR**	**95%CI**
IL-6	6.625	2.398-18.304	D-D >0.5	2.75	1.999-3.784
IL-8	6.881	2.453-19.298	D-D >1	3.733	2.241-5.756
AGE	4	1.493-10.714	D-D >2	16.5	6.832-42.656
D-D	16.5	6.382-42.656	D-D >4	29.4	7.148-120.9
NLR	4.6	1.242-17.08	D-D >8	55	6.523-463.7

### Kinetic analysis of WBC, NEU, LYM, MON, D-D dimer, and CRP in COVID-19 patients

**Figure 4:**
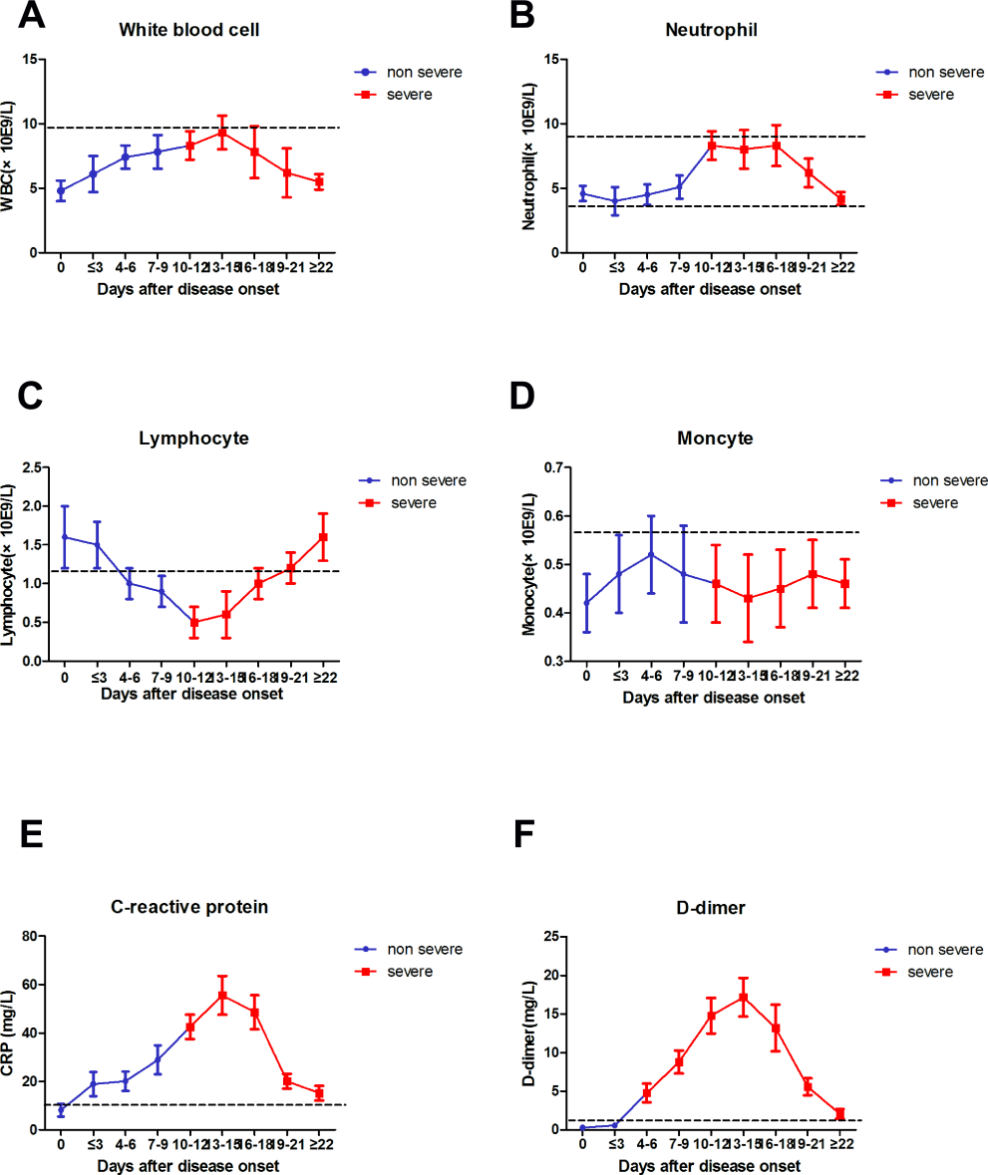
**Dynamic results of WBC, NEU, LYM, MON, CRP, D-D dimer from non-severe to severe.**

The absolute numbers of total WBCs (A), NEU (B), LYM (C), MON (D), CRP (E), and D-D (F) in the peripheral blood of mild (blue line) and severe (red line) COVID-19 patients were analyzed at different time points after hospital admission. Error bars represent mean ± SD. From non-severe to severe cases, the time for D-D to change from lower normal limit to upper normal limit was significantly earlier than that for other biomarkers, and the change was more evident (Figure 4).

## DISCUSSION

Since the outbreak of 2019-nCoV pneumonia in December 2019, the median incubation period was 4–7 days, and the fatality rate was relatively low [2]. By the end date of data collection (2020-02-29), less than 80,000 cases of COVID-19 were confirmed, and 2,835 patients died (CDC, China). The number of severe cases and deaths also increased every day [13]. In this study, severe case patients were older, and the proportion of underlying diseases was higher compared with mild case patients. Fever and cough were the first and most common symptoms before admission, whereas gastrointestinal symptoms, headache, shivering, pharyngalgia, and shortness of breath were rarely observed. This paper concluded the difference in viral tropism compared with SARS-CoV, MERS-CoV, and influenza [14, 15]. Fever occurred in 43.8% of patients upon initial presentation and developed in 83.4% after hospitalization. Among COVID-19, SARS–CoV (1%), and MERS–CoV (2%) infections, the absence of fever is most frequent in COVID-19 cases [14, 16], and the patients may be missed if the surveillance case definition focuses heavily on fever detection [3]. Significantly high frequencies of severe cases were observed in elderly patients with diabetes or hypertension (Tables 1 and 2). The clinical characteristics of these patients were similar to those reported in previous studies. [2, 3, 17]. From non-severe to severe, the time for D-D to change from lower normal limit to upper normal limit was significantly earlier than that for other biomarkers (Figure 4). Therefore, the D-D should be monitored every other day, which should also be beneficial for patients.

After the discussion of the clinical features of COVID-19, we analyzed the immunological characteristics of peripheral blood in patients with COVID-19. Firstly, the LYM counts were normal in COVID-19 patients with mild diseases [18]. By contrast, almost all patients with severe diseases had lymphopenia, and the LYM counts in patients with a mortal outcome remained at a low level [19]. This study also confirmed the higher rates of developing lymphopenia in severe case patients than in mild case patients (99.6% vs 67.2%). The development of lymphopenia in severe case patients was mainly related to the significantly decreased absolute counts of T cells, including CD4+ and CD8+T cells, similar to the absolute counts of B cells and natural killer cells but not to the proportion of LYMs. Interestingly, the ratio of CD4+ to CD8+ was mostly normal. This result is opposite that for acquired immunodeficiency syndrome. In addition, the immunoglobulin levels were normal, which may be explained by the acute infection caused by COVID-19 and half-life of immunoglobulin [20]. The number of T cells increased three to five days earlier than the relief of clinical symptoms. Thus, this course is associated with favorable outcomes among severe COVID-19 patients.

Elevated levels of proinflammatory cytokines, such as IFN, TNF-α, IL-6, and IL-8, are associated with severe lung injury and adverse outcome of SARS-CoV or MERS-CoV infection [20, 21]. Our results also demonstrated that severe COVID-19 patients had higher concentrations of IL-6, IL-8, IL-2,TNF-α, and IFN-γ in the serum than mild case patients, which suggested that the magnitude of cytokine storm was associated with the disease severity. The results indicated that COVID-19 may act on immune cells, especially T LYMs. T LYM damage is an important factor that causes patient deterioration. Additionally, T cells are important for dampening overactive innate immune responses during viral infection [22, 23]. Thus, the loss of T cells during COVID-19 may result in aggravated inflammatory responses, whereas restoration of T cell numbers may alleviate them. The courses of restoration of T cell numbers are associated with the decreases in serum IL-6, IL-10, IL-2, IL-4, TNF-α, and IFN-levels [24]. Therefore, the steady raise in the number of immune cells and the sustained decline in the levels of inflammatory factors are important laboratory manifestations for the clinical improvement of severe patients with COVID-19.

The decreased levels of immune cells and the increased number of inflammatory cells are important manifestations of COVID-19 infection. In the present study, the results supported the hypothesis which indicated that D-D dimer and elevated NLR are independent prognostic biomarkers affecting pneumonia progression in COVID-19 patients [25]. In addition, the integration of elevated NLR to prognostic nomograms may lead to improved prediction. The findings were consistent with those of previous studies on the relationship between NLR and prognosis of other infectious diseases [26]. The following reasons may account for the findings. On the one hand, NEU is a major component of leukocyte population that activates and migrates from the venous system to the immune organ or system. In addition, NEUs interact with distinct cell populations and produce numerous cytokines and effector molecules, such as circulating vascular endothelial growth factor. Furthermore, NEUs can be triggered by virus-related inflammatory factors, such as IL-6, IL-8, TNF-α, and granulocyte colony stimulating factor, and interferon gamma factors, which are produced by LYM and endothelial cells [27]. On the other hand, the increase in D-D is common in secondary hyperfibrinolysis conditions, such as hypercoagulable state, DIC, sepsis, and kidney disease [23, 28]. Hypercoagulable state blocks immune cell migration to infected organs and is incompatible with the novel coronavirus. Findings showed that the blocking factor is important given the doubled increase of D-D and the seven-fold increased risk ratio. Thus, infection-triggered inflammation increases NLR. Elevated NLR promotes COVID-19. The clinical symptoms become increasingly severe, and the progress from admission to intensive care unit, cure and discharge, or mechanical ventilation occurs rapidly. Thus, early identification of risk factors for severe COVID-19 patients may facilitate appropriate supportive care and prompt access to the intensive care unit if necessary.

In this study, the laboratory findings of acute renal function injury (4.94±1.69) appeared earlier than abnormal liver (7.81±1.86) and heart functions (6.19±1.83). However, several patients presented the opposite results. Further checking of electronic medical records revealed that these patients had hepatitis B infection and other common diseases, such as liver fibrosis, cyst, fatty liver, and cirrhosis. The causes are still unclear. Except for acute respiratory distress syndrome in patients caused by COVID-19, acute renal dysfunction may occur earlier than other organ dysfunctions, such as those of the liver and heart; these organs need to be monitored by clinicians. Hepatitis B infection is common in China [3, 29]; excluding this factor, whether we can arrive at the same conclusion remains to be further studied. Finally, PCA was performed to identify correlated variables for distinguishing severe and mild case patients (Figure 2). Five of the most contributing variables, namely, D-D dimer, IL-6, IL-8, age, and NLR, were selected as potential prognostic factors for further detailed statistical analysis. The optimal threshold of 3.3 for NLR indicated the superior prognostic possibility of clinical symptoms to change from light to heavy. D-D dimer had the highest sensitivity and specificity and the largest AUC.

Several notable limitations have been observed in this paper. First, the data were obtained from a single clinical research center. Second, the experimental data were limited to Han population of China. Furthermore, the conclusions of this study may differ from those of other scholars at home and abroad and must be further improved in clinical cases. Finally, accurate clinical data were lacking for the small number of patients with mild illness because of time constraints.

In conclusion, the study revealed that LYM subsets and cytokine profiles in the peripheral blood of COVID-19 patients were longitudinally characterized. D-D dimer increased from 0.5 to 8, and the risk ratio increased from 2.75 to 55, eventually leading to DIC. Acute renal function damage occurred earlier than the abnormal heart and liver functions. Finally, the kinetics features of immune parameters associated with the disease severity were determined, and D-D dimer and NLR were identified as the most useful prognostic factors for predicting severe COVID-19 cases.

## MATERIALS AND METHODS

### Patients

We performed a retrospective study on the clinical characteristics, epidemiological, demographic, laboratory data, and outcome data of laboratory-confirmed cases with 2019-nCoV. Cases were diagnosed based on the WHO interim guidance [5]; non-severe patients met all the following conditions: (1) epidemiological history, (2) fever or other respiratory symptoms, (3) typical computed tomography image of abnormities of viral pneumonia, and (4) positive result in reverse transcription polymerase chain reaction (RT-PCR) for SARS-CoV-2 RNA. Severe patients additionally met at least one of the following conditions: (1) shortness of breath, RR ≥ 30 times/min, (2) oxygen saturation (resting state) ≤ 93%, (3) PaO_2_/FiO_2_ ≤ 300 mmHg. Infections with other respiratory viruses, including influenza A virus, influenza B virus, respiratory syncytial virus and parainfluenza virus were excluded by serological test. Informed consent was waived in light of the anonymous, retrospective, and observational character of this study

### Clinical characteristics and laboratory data

The epidemiological characteristics (including recent exposure history), clinical symptoms and signs, and laboratory findings were extracted from electronic medical records. Laboratory assessments consisted of complete blood count, blood chemistry, coagulation test, liver and renal function, C-reactive protein (CRP), LYM subsets, and cytokines. The severity of COVID-19 was defined in accordance with the international guidelines for community-acquired pneumonia. The LYM test kit (FC 500 MCL, BECKMAN, USA) was used for LYM subset analysis (CD3+, CD3+CD4+, CD3+CD8+, CD16+CD56+, and CD3-CD19+). Plasma cytokines (interleukin (IL)-5, interferon (IFN)-α, IL-2, IL-6, IL-1β, IL-10, IFN-γ, IL-8, IL-17, IL-4, and IL-12P70) were detected with human Th1/2 cytokine kit II (ACEA NovoCyte, Guangzhou, China). All tests were performed in accordance with the product manual.

### Statistical analysis

Continuous variables were expressed as means and standard deviations or medians and interquartile ranges as appropriate. Categorical variables were summarized as the counts and percentages in each category. Wilcoxon rank-sum tests were applied to continuous variables. Chi-square tests and Fisher’s exact tests were used for categorical variables as appropriate. Optimal cutoff values of the continuous neutrophil (NEU)-to-LYM ratio (NLR), Age, D-D, IL-2, IL-6, IL-8, and CRP were calculated by applying the receiver operating curve analysis (ROC). Hazard risk and 95 % confidence interval were used as common measures to assess relative risk. Principal component analysis (PCA) was performed to identify the major contributing factors among clinical parameters to distinguish mild and severe cases of COVID-19 patients. P<0.05 was recognized as statistically significant. All statistical calculations were performed using SPSS 17.0 software (SPSS Inc, Chicago, USA).
